# Survey on the use of health services by adult men: prevalence rates and associated factors^1^

**DOI:** 10.1590/1518-8345.0296.2685

**Published:** 2016-03-28

**Authors:** Guilherme Oliveira de Arruda, Sonia Silva Marcon

**Affiliations:** 2 Doctoral student, Universidade Estadual de Maringá, Maringá, PR, Brazil. Scholarship holder from Coordenação de Aperfeiçoamento de Pessoal de Nível Superior (CAPES), Brazil; 3 PhD, Full Professor, Universidade Estadual de Maringá, Maringá, PR, Brazil

**Keywords:** Epidemiology, Men´s Health, Adult, Health Services, Socioeconomic Factors, Nursing

## Abstract

**Objective:**

estimate the prevalence and identify factors associated with the use of health services by men between 20 and 59 years of age.

**Method:**

population-based, cross-sectional domestic survey undertaken with 421 adult men, selected through systematic random sampling. The data were collected through a structured instrument and analyzed using descriptive and inferential statistics with multiple logistic regression.

**Results:**

the prevalence rate of health service use during the three months before the interviews was 42.8%, being higher among unemployed men with a religious creed who used private hospitals more frequently, had been hospitalized in the previous 12 months and referred some disease.

**Conclusion:**

the prevalence of health service use by adult men does not differ from other studies and was considered high. It shows to be related with the need for curative care, based on the associated factors found.

## Introduction

In knowledge production about man's health, studies have evolved towards solving the reduction on the health of the male in relation to the female population, reflecting on inequalities in health and appointing the importance of a broadened perspective on the differences between men and women regarding the morbidity and mortality profile^(^ 1 ^)^. A review on man's health indicates hypotheses to explain these differences: biological and genetic singularities of the sexes; social inequalities; gender differences; frailties in professional health care for men; little demand and use of health services by men^(^ 2 ^)^. These hypotheses support the characteristics of a background marked by unfavorable health conditions for men^(^ 2 ^)^.

The use of health services is a complex behavior with multifaceted determinations, according to differences in locations, socioeconomic situation, morbidity profiles, quality of life and knowledge about health^(^ 3 ^)^. In a study about factors associated with health service use, important variations are revealed according to age, sex, income, education, skin color and even differences inherent in the type of care model that is adopted^(^ 4 ^-^ 5 ^)^. In that context, health surveys can provide important information on the characteristics of the population, which are necessary to formulate practices and public policies, considering that health services particularly avail themselves of the users' problems to elaborate their actions^(^ 6 ^)^.

The groups of men who use health services are not homogeneous and, therefore, international recommendations appoint the need for studies that demonstrate these differences in the profiles, so as to support alternative approaches to man's health^(^ 7 ^)^. Studies on the use of health services are undertaken to identify what factors are associated and what determines the population, in this case adult men's use of the services. Knowledge on these factors can equip professionals and managers to plan, operate and assess care strategies, programs and models^(^ 8 ^)^, with a view to promoting the supply of and access to the services, entailing improvements in the health profile of the male population.

In that sense, the objective in this study was to estimate the prevalence and identify the factors associated with the use of health services by adult men, according to socioeconomic, demographic, health and service usage characteristics.

## Method

A population-based, cross-sectional domestic survey was undertaken, involving male individuals between 20 and 59 years of age living in the city of Maringá, PR, Brazil. The data were collected between January and July 2013, mainly during weekdays, in the morning and afternoon. The male population in the city between 20 and 59 years of age corresponded to 103,819 (60.2% of the male population in the city), according to the 2010 Demographic Census. Based on criteria by the Brazilian Institute for Geography and Statistics, such as the participation of family heads in the job market, the city's territory is divided into 20 Weighting Areas.

To select the participants, the systematic random sampling technique was used and the only eligibility criterion was being between 20 and 59 years of age. The men were contacted at home, in accordance with the subsamples proportional to the number of people in each Weighting Area. Based on the number of eligible individuals, the streets to be visited were randomly drafted. Thus, at preset intervals, a male dweller was contacted, starting with the fourth house on the right side of the street. If no individual who complied with that inclusion criterion existed/was present, respecting the preset interval, the next house was visited, with a view to avoiding losses due to a lack of respondents.

The minimum sampling size was defined based on the following parameters and estimates: 50% for the prevalence of the event of interest, associated with a 5% estimation error and 95% reliability of the sample. Ten percent (38 individuals) was added to the minimum sample calculated (383 individuals), considering the possibility of losses in the completion of the data collection tools. The study sample consisted of 421 individuals.

The response variable "use of the health service" was verified by means of the question "When did you last visit a health service?". For analysis purposes, the three-month period before the interview was adopted to reduce the memory bias in the answers beyond that interval (underestimation of prevalence), besides permitting comparisons with other studies that intended to analyze the same outcome, also considering the three-month period before the interview^(^ 6 ^,^ 9 ^)^.

The independent variables were the socioeconomic and demographic characteristics: age range, skin color, marital situation, education, children, religion, work, family income, occupational status, health insurance and economic class, analyzed according to the Brazilian Economic Classification Criterion of the Brazilian Association of Research Companies, which considers the education level of the family head and the ownership of certain domestic items, as used in another study on the use of health services^(^ 10 ^)^. The service usage and health variables were: type of service, care and management; reason for visit, hospitalization in the previous 12 months, self-perceived health (perception of own health, using the health of other people of the same age for reference) and referred morbidity.

The data were collected through interviews, using a structured tool the researchers had elaborated. Before the compilation in a database in Microsoft Office Excel 2010, the researchers checked the completed tools for errors. Next, the database was transferred to IBM Statistical Package for the Social Sciences (SPSS), version 20.

The data were analyzed using descriptive and inferential statistics, divided between two moments: gross (univariate) analysis using Pearson's non-parametric chi-square test, in which all independent variables were tested, and adjusted (multiple) analysis using non-conditioned Multiple Logistic Regression Models. At this second moment, the model was adjusted based on the literature^(^ 11 ^-^ 12 ^)^, using the following sociodemographic variables: age range, skin color, marital situation and education. In addition, the forward method was applied, through which the variables with p<0.20 in the gross analysis were inserted in the logistic model according to the decreasing order of the p-value, so as to gradually verify the significance variations, besides the maintenance or exclusion of variables from the model. The Odds Ratio (OR) was used as the association measure, with a 95% confidence interval and significance was set at p<0.05 for the tests applied.

The development of the study complied with Brazilian ethical recommendations for research involving human beings, as recommended by the National Health Council. Approval for the project was obtained from the institution's Ethics Committee (CAAE: 10754612.2.0000.0104).

## Results

Among the 421 individuals interviewed, a considerable part was between 40 and 49 years of age (28%), had taken up to secondary education (36.8%), gained between 2.1 and 4 minimum wages (34%) and was an employer/self-employed (40.9%). The majority was white (58%), had a partner (67.9%), children (71.3%), a religious creed (89.8%), active in the job market (80.3%), had a health insurance (52.7%) and fit into economic class B (53%).

The prevalence of health service use during the three months before the research was 42.8% (n=180) and was higher for the public services (57.8%). The most used service type was the primary health care service (46.3%), the most used type of attendance was the doctor's appointment (82.7%) and disease was the most prevalent reason to visit the service (55.3%). During the previous 12 months, 11.7% of the men had been hospitalized. Most men (77.0%) showed a positive self-perceived health, although 42.8% reported some morbidity.

In Table 1, it is observed that, in the univariate analysis, "occupational status" and "work" were associated with health service use in the three months preceding the research.

**Table 1:** - Distribution and univariate analysis of health service use by adult men in the last three months according to socioeconomic and demographic variables. Maringá, PR, Brazil, 2013

**Socioeconomic and demographic variables**	**Health service use**			
	**n**	**%**	**OR (95%CI)**	
Has children				
	No*	50	41.3	1 1.08 (0.70; 1.66)
	Yes	130	43.3	
Religion^†^				
	Yes*	157	41.5	1 1.11 (0.96; 1.30)
	No	23	53.5	
Work^‡^				
	No*	45	54.2	1 0.60 (0.35; 0.91)
	Yes	135	39.9	
Family income				
	Up to 2*	31	43.7	1
	2.1 to 4	67	46.9	1.13 (0.64; 2.01)
	4.1 to 6	34	35.8	0.72 (0.38; 1.35)
	More than 6	48	42.9	0.97 (0.53; 1.76)
Occupational status^‡^				
	Employer/self-employed	71	41.3	1
	Employee	57	36.3	0.81 (0.52; 1.26)
	Retired/on medical leave	31	66.0	2.75 (1.40; 5.41)
	Unemployed	14	70.0	3.31 (1.21; 9.05)
	Student/trainee	7	28.0	0.55 (0.22; 1.39)
Health insurance^†^				
	No*	93	38.9	1 1.37 (0.93; 2.03)
	Yes	86	46.7	
Economic class				
	Class A*	13	41.9	1
	Class B	92	41.3	0.97 (0.45; 2.08)
	Class C	69	43.9	1.08 (0.50; 2.37)
	Class D	5	55.6	1.73 (0.39; 7.72)

*Reference category; †p<0.20; ‡p<0.05

According to Table 2, the univariate analysis revealed that the health service use was associated with the service demand for the sake of rehabilitation, hospitalization in the previous 12 months, negative self-perceived health and referred presence of morbidity.

**Table 2:** - Distribution and univariate analysis of health service use by adult men in the last three months according to usage and health variables. Maringá, PR, Brazil, 2013

**Usage and health variables**	**Health service use**			
	**n**	**%**	**OR (95%CI)**	
Service type				
	Primary health care service*^†^	78	40.0	1
	Outpatient clinic	6	54.5	1.80 (0.53; 6.10)
	Emergency care^‡^	23	38.3	0.93 (0.51; 1.69)
	Hospital	20	50.0	1.50 (0.76; 2.96)
	Others	53	46.1	1.28 (0.80; 2.04)
Type of attendance				
	Medical consult*	150	44.2	1
	Vaccination and nursing care	9	32.1	0.59 (0.26; 1.35)
	Dental care	13	43.3	0.96 (0.45; 2.04)
	Others	8	61.5	2.01 (0.64; 6.29)
Type of management ^§^				
	Public*	95	40.1	1 1.44 (0.97; 2.14)
	Private/health insurance	85	49.1	
Motive^||^				
	Accident/injury*	9	28.1	1
	Rehabilitation	7	87.5	17.9 (1.91; 166.8)
	Vaccination and other preventive care	46	43.0	1.92 (0.81; 4.55)
	Disease	105	45.1	2.09 (0.93; 4.72)
	Dental care	12	42.9	1.91 (0.65; 5.61)
Hospitalization in the last 12 months^||^				
	No*	148	39.9	1 2.83 (1.52; 5.29)
	Yes	32	65.3	
Self-perceived health^||¶^				
	Positive*	125	38.6	1 2.08 (1.31; 3.30)
	Negative	55	56.7	
Morbidity^||^				
	No*	77	32.0	1 2.85 (1.90; 4.25)
	Yes	103	57.2	

*Reference category. †Primary Health Care Service; ‡Emergency care; §p<0.20; ||p<0.05; ¶positive: excellent/very good/good; negative: regular/bad/very bad

Table 3 displays the variables that remained associated with the health service in the adjusted multiple logistic model. Greater proportions of health service use were observed among unemployed men, with a religious creed, who used private or health insurance service, had been hospitalized in the previous 12 months and referred at least one health problem.

**Table 3:** - Multiple logistic regression model of factors associated with health service use by adult men in the last three months. Maringá, PR, Brazil, 2013

Associated factors *	**OR (95%CI)**	
Occupational status		
	Employer/self-employed^†^	1
	Employee	0.77 (0.47; 1.25)
	Retired/on medical leave	1.70 (0.79; 3.65)
	Unemployed	3.06 (1.06; 8.86)^‡^
	Student/trainee	0.58 (0.20; 1.65)
Religion		
	No^†^	1
	Yes	2.05 (1.01; 4.17)
Type of management		
	Public^†^	1 2.21 (1.36; 3.61)^‡^
	Private/health insurance	
Hospitalization in the last 12 months		
	No^†^	1 2.46 (1.24; 4.85)^‡^
	Yes	
Morbidity		
	No^†^	1 2.33 (1.47; 3.68)^‡^
	Yes	

*Model adjusted by the variables "age range", "skin color", "marital situation" and "education"; †reference category; ‡p<0.05

## Discussion

Brazilian studies that estimated the prevalence of health service use by adults simultaneously studied men and women and differ in terms of the health service usage period before the interview and the type of service and care used^(^ 6 ^,^ 9 ^)^. Hence, health service use by the male population should be addressed with caution and weighting in view of the particularities discussed in the literature. In addition, the exclusive focus on the male adult population is highlighted as a distinctive and important aspect for knowledge in male health.

The prevalence found did not differ much from other studies, despite the methodological differences. Population-based surveys undertaken in Pelotas found prevalence rates of 42.6% in 2003^(^ 9 ^)^ and 35.1% in 2008^(^ 6 ^)^ for health service use among men during the three months prior to the interview. Nevertheless, the first only considered the doctor's appointments, without restrictions as to the type of health system management (public or private), and the second considered only doctor's appointments in the public system. The 2008 National Household Survey, in turn, considered the use of public and private services by men between 20 and 64 years of age during the 14 days before the data collection, and found a prevalence rate of 21.8%, much lower than the rate of women in the same period (38.7%)^(^ 13 ^)^.

Although the literature appoints that women use health services more often, mainly due to gynecological, genital-urinary and obstetric causes and greater perceived health risks^(^ 9 ^)^, it is highlighted that health service use has increased in general, thanks to the expanded coverage of the Unified Health System (SUS), greater access to private health insurances and health information^(^ 3 ^)^. The prevalence of mean who used services offered by the SUS (public) is similar to that observed in a study undertaken in homes all over Brazil in 2008, amounting to 56.3%. Nevertheless, it is important to highlight that, on the one hand, the period before the interview was shorter (previous 14 days), on the other, individuals aged 15 years or older were contacted, including elderly people^(^ 5 ^)^. As health services tend to be used more among the elderly^(^ 8 ^)^, their participation tends to increase the prevalence, even when a shorter period is considered.

As regards the services men use more, during the three months before the research, it was verified in a study undertaken in the city of Pelotas that the prevalence of primary health service use among men and women corresponded to 49.5%, without significant differences between the sexes^(^ 6 ^)^. Therefore, the prevalence found in this study is not distant from that found in the other research. In view of the logic of the high population coverage, expanded access and service organization based on integral health care, the relevance of primary health care services is highlighted as the services the male population uses most^(^ 3 ^)^.

The higher proportional use of this service, however, should not be the sole parameter to verify the actual access to public services nor the attendance to the male population's health needs as, according to the literature, it is common for men to enter the health system through specialized care services, due to the worsening of a disease, deriving from a delay to seek care^(^ 1 ^)^. In a study developed in Ribeirão Preto, SP, the duplication of services was found, that is, the simultaneous use of primary health care and emergency services for the same end (diagnosis, treatment or rehabilitation), pointing towards the absence of problem-solving care and dissatisfaction among the users^(^ 14 ^)^.

Aiming for integral care, therefore, the need emerges to create conditions to produce care at all levels, but mainly in Primary Care, in which the teams frequently face a lack of time for planning, structural shortages in the services and professional limitations in the interventions^(^ 15 ^)^.

The literature appoints that the doctor's appointment is the main type of care demanded. Some studies exclusively focus on the usage pattern of this type of care^(^ 4 ^,^ 6 ^,^ 9 ^)^. Concerning men's demand for doctor's appointments, in a review on the health situation of the male population in Canada, 80% of the men refuse to consult the doctor and only give in when convinced by their wife or partner^(^ 16 ^)^. Nevertheless, the higher proportional use of medical consultations is not only due to men's demand for this type of care but is also the services' main response to the population's health problems, of rationality mainly centered on a curative care model that only privileges the consumption of the service offered, secularizing and abolishing other care possibilities.

In terms of factors admittedly associated with the use of health services, it should be highlighted that the literature has appointed the importance of the relation between advanced age and increased frequency of health service use, due to the new care demands, more frequent hospitalization and longer/more expensive interventions, as consequences of aging associated with illness^(^ 8 ^,^ 17 ^)^. These findings underline the premise that the age range should be considered, like in this study, as an adjustment factor to estimate explanatory models for the use of health services. Nevertheless, it is highlighted that, in the preliminary univariate analysis (without theoretical adjustments), the age range showed no statistically significant association, possibly due to the fact that, among men between 20 and 59 years of age, the proportion of health service use did not vary among the age ranges, as opposed to what could have been observed if elderly men were included in the study. In addition, the interval between 50 and 59 years, which showed a greater difference when compared to the reference category (20 to 29 years), was not the most frequent in the total sample, leading to the conclusion that the sample size of the age subgroups may have influenced the non-identification of an association between age and health service use.

The greater use of health services by unemployed individuals, as verified in this study, was also identified in the population covered by the family health strategy in Porto Alegre, RS^(^ 3 ^)^. In another study, developed in ten Brazilian metropolitan regions, an association was found between unemployment and worse health conditions among adult men between 15 and 64 years of age^(^ 18 ^)^. The work relations result in health inequalities, demanding the implementation of effective policies and institutional changes to promote equity^(^ 18 ^)^. Hence, besides overcoming barriers, increasing the service supply and adapting the health institutions' profile to the users who work, attention to unemployed individuals is urgent, especially in the adult male population^(^ 18 ^)^, for which work indicates the exercise of manhood.

Having a religious creed was associated with greater health service use among the investigated men. Hence, adhering to or following a religion influences, sometimes positively, the health conditions and the adoption of healthy behaviors. A study involving 345 adult Latin individuals living in Texas showed that the proportions of alcohol abuse and smoking were significantly higher among people who indicated having no religion at all^(^ 19 ^)^. In another study involving 1,076 North Americans, it was shown that having a religion predisposes to greater health service use for preventive ends, mainly because they participate in preventive activities in the religious community, and also because they talk about behaviors and tests with peers who follow the same religion^(^ 20 ^)^.

Paradoxically, an association was found between the occupational status "unemployed" and the management type "private" and health service use. Nevertheless, this can be justified as follows: 35% of the unemployed men informed having a health insurance, some of whom informed they were registered as dependent in their partner or children's insurance so that, even without an income, they had access to health insurance services; 30% of the people who indicated they were unemployed were between 20 and 29 years old, that is, young men, who were not even professionally active yet, with studying as their main activity, and therefore dependent on the family for expenses in general and health expenses in particular. Even in case of unemployment, private health services can be used when other family members pay for them.

The most frequent type of health system gained significance in the multiple analysis, like hospitalization in the previous years and reference to some disease. When the use per management type is verified, a greater demand for health services is observed for individuals who use private health services, despite the increased demand for public services in the general population. One aspect that was not mentioned in this study refers to the use of public services by insured people/people with a health insurance as a secondary option, mainly in function of some services charged in the private sphere, like vaccination, or delivered exclusively in the public sector, like medication distribution^(^ 21 ^)^. These two types of services frequently attract men to the public health system^(^ 22 ^)^.

Concerning hospitalization, the prevalence of this event was much higher than in a Brazilian study using PNAD data, in which the prevalence of hospitalization amounted to 7.0 and 7.1%^(^ 18 ^)^ in 2003 and 2008, respectively, and also higher than in a study in the South of Brazil^(^ 9 ^)^. Hospital morbidity is an important indicator in the analysis of the male population's health profile, which should be considered in the coordination of health care networks and in health service organization. That is so because many groups of causes associated with hospitalizations among adult men are linked with harmful health behaviors and are causes avoidable through primary health care, in line with an ecological study that used data from the city of Maringá, PR^(^ 23 ^)^.

Hospitalization in the previous year and the presence of morbidity played a determinant role in health service use during the three months prior to the research. This finding was also evidenced in two studies developed in Pelotas, RS, among adults, highlighting a greater proportion of doctor's appointments among men hospitalized within one year before the interview^(^ 6 ^,^ 9 ^)^. This finding may be related to the fact that the individuals who were hospitalized are sicker than people who were not hospitalized and, therefore, have a greater demand for health services^(^ 9 ^)^. In addition, adult men tend to seek health care only when their health condition gets worse or when they are confronted with the need for hospitalization. In view of the results, it is suggested that the health services' care coverage, mainly in the family health strategy, is a determinant factor in the monitoring of the individuals covered, and potentially in the reduction of hospitalizations.

The prevalence of positive self-perceived health was very similar to the findings in studies involving adults in general, undertaken in Pelotas, RS (77.7%)^(^ 24 ^)^. Self-perceived health and referred morbidity are important indicators of the population's burden of health problems and can also be used as indicators of health needs concerning the use of different types of health services^(^ 25 ^)^, although self-perception lost significance in the multiple analysis applied in this study, like in another study^(^ 6 ^)^. In addition, the prevalence of referred morbidity remained within the limits evidenced in a Brazilian study in the age groups between 25 and 49 years (33.9%) and between 50 and 64 years (62.0%)^(^ 26 ^)^.

It was verified that the referred morbidity was associated with greater use of health services among adult men. This finding was in line with the literature, mainly concerning chronic illnesses^(^ 9 ^,^ 10 ^,^ 27 ^)^. This appoints that men are seeking monitoring, which is positive as, sometimes, they are known for the difficulty to socialize their health problems and, after conquering this barrier, the health professionals have the opportunity to establish a therapeutic bond. On the other hand, it may indicate that they only seek professional support when they are ill. This is a source of concern, especially for young men, who should spontaneously seek health promotion and preventive care orientations as well, beyond mere medical and curative care.

Having a health insurance was not identified as an associated factor, in turn, although the literature appoints that this resources allows men to use health services more frequently, as evidenced in a study among Asian and American men^(^ 28 ^)^. Even when they live in an area covered by the family health strategy, adult men can choose to use health insurance services^(^ 6 ^)^ for reasons like the delay to get care, the lack of professionals and inputs, the predominance of women, children and elderly people at the primary care services, lack of valuation of preventive practices and fear because they cannot choose the professional who will attend them^(^ 22 ^)^. Therefore, having a reference professional can play a determinant role in the demand for and use of the health services^(^ 6 ^)^. In a population-based study, for example, it was observed that the probability of having a prostate exam increased by 98% among patients who indicated having a reference professional^(^ 4 ^)^.

Some methodological limitations should be weighted, such as the non-stratification of the sample per age range or by occupational status, the use of a non-validated data collection tool and the collection of self-referred information, subject to memory lapses. In addition, the time and the days on which the data were collected may have been a limiting factor for men who work during office hours, although many participants were interviewed at lunchtime. Hence, these are challenges for future research.

Despite the limitations, this kind of studies should be encouraged, as the results found are not only valid and comparable to other studies, but grant knowledge on health service use among adult men in specific contexts. Their findings can contribute to clarify the factors that predispose to this behavior, with a view to the reorganization of the services and the implementation of actions to get closer to, welcome and deliver integral care to the male population.

## Conclusion

In this study, it is shown that the prevalent use of health services among adult men (42.8%), although similar to other studies, was considered high. Besides investigating the use of public and private services, only men up to 59 years of age were included. Concerning the associated factors, the results are in accordance with the literature, but studies that investigated these factors specifically in the male population remain scarce.

The findings on the association between unemployment and use of private or health insurance plans stand out, based on which it can be inferred that the access to these types of services derives from family dependence, especially when considering that most of the men who self-declared they were unemployed were between 20 and 29 years of age. Thus, in the current family configuration patterns, young people commonly stay longer at their parents' home and depend on them, also for health expenses. Another issue that should be considered is that, although many consider they are family heads and unemployed, another person can serve as a family provider and pay for the family's needs.

In view of the associations found, mainly factors like hospitalization in the previous 12 months, negative self-perceived health and reference to some morbidity, health service use among adult men seems to be related with the need for curative care. Therefore, it is fundamental that, from the formulation to the implementation of man's health policies and programs, the associations found in this study are taken into account.
